# Intersectional Stigma and Implementation of HIV Prevention and Treatment Services for Adolescents Living with and at Risk for HIV: Opportunities for Improvement in the HIV Continuum in Sub-Saharan Africa

**DOI:** 10.1007/s10461-022-03793-4

**Published:** 2022-07-30

**Authors:** Lonnie Embleton, Carmen H. Logie, Kenneth Ngure, LaRon Nelson, Liza Kimbo, David Ayuku, Janet M. Turan, Paula Braitstein

**Affiliations:** 1 Dalla Lana School of Public Health, University of Toronto, 155 College Street, Toronto, ON M5T 3M7 Canada; 2 Factor-Inwentash Faculty of Social Work, University of Toronto, Toronto, ON Canada; 3 School of Public Health, Jomo Kenyatta University of Agriculture and Technology, Nairobi, Kenya; 4 School of Nursing, Yale University, Orange, CT USA; 5 School of Public Health, University of Alabama at Birmingham, Birmingham, AL USA; 6 Department of Behavioural Sciences, Moi University, College of Health Sciences, Eldoret, Kenya; 7 College of Health Sciences, School of Public Health, Moi University, Eldoret, Kenya; 8 Academic Model Providing Access to Healthcare (AMPATH), Eldoret, Kenya

**Keywords:** Intersectional stigma, Adolescents, HIV, Implementation science, Sub-Saharan Africa

## Abstract

Adolescents in sub-Saharan Africa, specifically adolescent girls and young women, young men who have sex with men, transgender persons, persons who use substances, and adolescents experiencing homelessness experience intersectional stigma, have a high incidence of HIV and are less likely to be engaged in HIV prevention and care. We conducted a thematic analysis informed by the Health and Discrimination Framework using a multiple case study design with five case studies in 3 sub-Saharan African countries. Our analysis found commonalities in adolescents’ intersectional stigma experiences across cases, despite different contexts. We characterize how intersectional stigma impacts the uptake and implementation of HIV prevention and treatment services along the continuum for adolescents. Findings reveal how intersectional stigma operates across social-ecological levels and worsens HIV-related outcomes for adolescents. We identify opportunities for implementation science research to address stigma-related barriers to the uptake and delivery of HIV services for adolescents in sub-Saharan Africa.

## Introduction

Adolescents in sub-Saharan Africa are one of the highest affected groups globally in terms of HIV [1–3], yet, not all adolescents are affected equally. Adolescent girls and young women (AGYW), specifically, continue to have high HIV incidence, as do adolescent boys and young men who have sex with men (YMSM), transgender persons, persons who use substances, and adolescents experiencing homelessness [4–6]. Adolescents broadly are less likely to be tested for HIV compared to adults, less likely to access HIV treatment, and less likely to make optimal use of various HIV prevention methods, both behavioral and biomedical [5, 7, 8]. AGYW and other adolescent key populations are particularly challenged across the HIV prevention-care continuum [9–13]. A common element affecting HIV service use among adolescents is stigma [14–16]. Stigma impacts populations at risk for and living with HIV in myriad ways, causing delays in accessing HIV preventive information and services (including condoms and lubricant), uptake of HIV testing and treatment services, and ultimately stigma and its impacts negatively influence HIV outcomes [17–21].

Stigma is a social determinant of health and refers to social processes of devaluation that result in reduced power and opportunity for the stigmatized group [14, 22, 23]. Stigma occurs across all socio-ecological levels (structural, community, organizational, interpersonal, and individual) [17, 24]. The processes and manifestations of stigma are influenced by social (e.g., cultural and gender norms) and structural (e.g., policies and laws) factors in a given context [17]. For example, social–cultural norms across many sub-Saharan African contexts facilitate adolescent sexual and reproductive health stigma related to sexual activity outside of marriage, unintended pregnancy, and use of contraception, and influence the uptake and delivery of sexual and reproductive health services, particularly in association with HIV [8, 14, 25–28]. Likewise, across political contexts in many sub-Saharan African countries, laws and policies criminalize same-sex sexual relations, and facilitate the process of stigmatizing YMSM, and ultimately impact HIV-related outcomes for this group [29–31].

HIV stigma encompasses the attitudes and behaviors that devalue individuals based upon their actual or perceived status, or their loved ones’ HIV status, and this can negatively affect engagement in HIV services [22, 32]. Adolescents living with and at risk for HIV negotiate HIV stigma in their daily lives, and often have multiple intersecting identities that may amplify experiences of HIV stigma, reflecting in what is described as ‘intersectional stigma’ [24, 33–35]. Intersectional stigma is a concept that can be used to understand the convergence of multiple stigmatized identities within a person or group and their effects [33]. These intersecting identities may include those based on ethnicity, gender and gender identity, sexual orientation, age, class, substance use, mental health, homelessness, among others. The intersection of these social identities may amplify experiences of privilege or oppression [24, 33]. An intersectional stigma lens can enable researchers and others to consider how multiple stigmatized identities impact health behaviours and outcomes [33].

Intersectional stigmas can be experienced as awareness of negative community norms and beliefs (perceived community stigma, or stigmatising lay attitudes); acts of mistreatment and discrimination, including in employment, education, and healthcare (enacted stigma); negative self-image, shame, and blame from larger contexts of mistreatment and exclusion (internalised stigma); and fear and expectations of experiencing devaluation, exclusion, and discrimination when others learn about their stigmatised practice/identity (anticipated stigma) [14]. Intersectional stigma manifests at various stages of the HIV prevention-treatment continuum for adolescents in sub-Saharan Africa in complex ways and thus influences the implementation and uptake of feasible, acceptable, effective, and sustainable HIV services and interventions.

Several highly effective evidence-based interventions, practices, and policies exist to improve outcomes across the HIV prevention-treatment continuum for people living with and at risk of HIV [36–39]. However, the implementation and uptake of these evidence-based interventions, practices, and policies for adolescents living with or at risk of acquiring HIV in some sub-Saharan African contexts has been limited, in part due to stigma associated with adolescent sexual and reproductive health and HIV [40–44]. Implementation science research, concerned with the research to practice gap, *is the study of methods to promote the adoption and integration of evidence-based practices, interventions, and policies into routine health care and public health settings to improve our impact on population health* [45, 46]*.* Context is an important implementation determinant and can be defined as the set of circumstances or unique factors that influence an implementation effort in a specific setting [47]. Intersectional stigma may act as a contextual determinant to the uptake and implementation of HIV prevention and treatment services for adolescents living with or at risk of acquiring HIV. Implementation science research offers tools to optimize adolescent HIV prevention and care and address stigma as a barrier to the uptake and delivery of HIV services for adolescents along the HIV prevention-treatment continuum [38, 42].

This paper will apply an intersectional stigma lens to five case studies illustrative of the lived experiences of adolescents in three sub-Saharan African countries, representing a multiplicity of social identities and complex realities encountered in our authors’ clinical and research experiences. We will use these case studies as exemplars to characterize how intersectional stigma and elements of socio-cultural, organizational, and political-economic contexts impact the uptake and delivery of HIV prevention and treatment services, and how intersectional stigma, together with other individual, interpersonal, and structural determinants, can be targeted for change. We apply the Health Stigma and Discrimination Framework to examine the drivers and facilitators of intersectional stigma and assess how it manifests in stigma experiences and practices among adolescents in healthcare and community settings across the five cases from three sub-Saharan African countries [17]. We use these case studies to propose opportunities for implementation science research to address stigma-related barriers to the uptake and delivery of HIV services and improve engagement by adolescents along the HIV prevention-treatment continuum.

## Methods

### Study Design

This study uses a multiple-case design [48], where several cases were selected to develop an in-depth understanding of the phenomenon of intersectional stigma experienced by adolescents living with or at-risk of acquiring HIV in sub-Saharan Africa. Through examining processes and outcomes across cases and exploring how cases may be affected by different contexts and conditions, the multiple-case design facilitates a rich understanding of an issue. This study design allowed us to identify key opportunities for implementation science research to improve the uptake and delivery of HIV services along the care-continuum for adolescents in sub-Saharan Africa.

### Data Sources

We generated five cases based on data drawn from multiple studies and sources among the author team, which included quantitative, qualitative, informal reports from adolescents and youth, and observational data from clinicians and researchers working with adolescents. Data from these studies and sources was organized into five narratives that explore stigma and discrimination experienced by adolescents living with and at risk of acquiring HIV in sub-Saharan Africa. Table 1 gives an overview of the cases and summarizes the original studies and sources of data. The 5 narratives were created from data collected from studies that focused on the adolescent population highlighted in each case (e.g., pregnant adolescent girl living with HIV, orphaned adolescent girl, street-connected adolescent girl, refugee girl, and young MSM). Within each case we explore how intersectional stigma acts as a contextual determinant of implementation using the Health and Discrimination Framework. The original studies and sources of data that contributed to generating the 5 cases either measured stigma quantitatively, explored facets of intersectional stigma qualitatively, or offered opportunities for collecting informal reports from adolescents and observational data from clinicians and researchers. The findings related to stigma from these studies then informed the development of the narratives by the authors. A methodological description of the cases and their data are described below.

**Table 1:** Summary of case studies and data sources

	Case A	Case B	Case C	Case D	Case E
Adolescent key population highlighted in case study and	Pregnant adolescent girl living with HIV at-risk of intimate partner violence	Street-connected adolescent girl	Orphaned adolescent girl	Young refugee woman	Young MSM and partner
Age range of study participants from original sources of data	Range: 18–45 years	Range: 15–24 years	Range: 14–19 years	Range: 16–24 years	Range: 18–55 years
I Stage(s) in HIV prevention-care continuum	HIV testing and counseling; linkage to and engagement in care; PMTCT	HIV testing and counseling	HIV testing and counseling; HIV prevention	HIV testing and counseling; HIV prevention (post-exposure prophylaxis)	Retention in care /transition to adult care; HIV prevention (PrEP)
Intersectional stigmas	HIV status, poverty, gender, age, intimate partner violence	Homelessness/street identities, poverty, gender, age, and sex work	Adolescent sexuality, gender, age, orphan status, poverty	Sexual violence, refugee status, gender, age, and HIV	Sexuality, age, gender norms, HIV
Geographical setting	Rural southwestern Kenya	Uasin Gishu, Nakuru, Trans Nzoia, Bungoma, and Kisumu counties; Eldoret, Kenya	Uasin Gishu County, Kenya	Bidi Bidi settlement, rural area in the Northern Region, Uganda	Accra, Kumasi and Manya Krobo communities in Ghana
Key contextual factors influencing stigma	Social–cultural: Tolerance of IPV, violence associated with disclosure of positive HIV status, poor awareness of legal rights, lack of social/community support, HIV stigma, polygyny Political: Laws and policies on IPV Economic: Economic dependency on male partners, poverty	Social–cultural: adolescent sexuality framed as taboo; street-involvement and sex work highly stigmatized in social-cultural context Political: Laws and policies concerning street-involvement Economic: Extreme poverty, economic dependency on sex work	Social–cultural: Adolescent sexuality framed as taboo Political: Laws challenging access to SRH services for adolescents Economic: extreme poverty	Social–cultural: adolescent sexuality framed as taboo; very rural so confidentiality concerns for HIV services Political: conflict affected youth have trauma and SGBV histories; limited access to prevention resources such as lubricant and PrEP Economic: extreme poverty, high unemployment	Social–cultural: Social norms are generally anti-LGBT; conservative religious beliefs have dominance in everyday Ghanaian life Political: Intense public policy debates regarding whether constitution offers equal legal protection of sexual minorities Economic: extreme poverty, high unemployment
Stigma manifestations across social-ecological	Individual, interpersonal, community, organizational	Individual, interpersonal community organizational	Individual, organizational, structural	Individual, interpersonal, community	Interpersonal, organizational, structural
Summary of data sources from studies informing cases	1. Mixed methods study on role of HIV-related stigma in utilization of child-birth services. [49] 2. Mixed methods study on IPV and forced migration IPV for pregnant women. [50] 3. Qualitative study on facilitating HIV disclosure by pregnant women and partners. [51] 4. Qualitative sub-study on self-disclosure and resistance to HIV stigma. [52]	1. Qualitative study of community perceptions of SCY. [53–55] 2. Qualitative study of SRH practices among SCY. [56–59] 3. Pilot intervention engaging SCY in HIV prevention [61–63] 4. Pilot intervention engaging SCY in HIV prevention-care continuum [60]	1. Longitudinal cohort of orphaned and separated children and adolescents (OSCA) in western Kenya [63–65] 2. Clinical experience with adolescents and SRH needs [67–69]	1. Qualitative study focused on sexual and gender-based violence prevention and post-rape clinical care with refugee adolescents and youth in Bidi Bidi, Uganda [70, 72]	1. Mixed methods (qualitative description and quantitative description) study of 22 peer social networks of MSM in Ghana to understand the factors influencing HIV prevention and risk behaviors [73–76]
Summary of study participants from original data source(s)	1. Pregnant women recruited at ANC (n = 1777), IDIs with postpartum women, traditional birth attendants, CHWs, and family members (n = 48) 2. Pregnant women recruited at ANC (n = 614), IDIs and FGDs with pregnant women, male partners, and service providers (n = 123) 3. IDIs with HIV+ women (n = 20) and male partners (n = 20). FGDs with service providers (n = 16) 4. IDIs with HIV+ pregnant women and male partners, both sero-concordant and discordant (n = 38)	1. SCY aged 15–24 years (n = 43, 3 FGDs and 6 IDIs), healthcare providers, policymakers, police officers, community members, SCY stakeholders (n = 54, 4 FGDs and 35 IDIs) 2. SCY aged 11–24 years (N = 65, 5 FGDs, 25 IDIs) 3. SCY aged 24 years (N = 80, 4 FGDs, pilot observational notes) 4. SCY aged 0–24 (N = 781, pilot observational notes)	1. OSCA aged < 19 years at enrolment living with extended family, in orphanages and rescue centers, on the street	1. Focus groups and in-depth interviews with refugee adolescents and youth aged 16–24 years old living, elders, and health care providers in Bidi Bidi [70, 72]	1. Focus groups, interviews and quantitative surveys, with MSM (N = 137) ages 18–55 (M = 25.65, SD = 5.4) living in Accra, Kumasi and Manya Krobo [73–76]

*IDIs* in-depth interviews; *FGDs* focus group discussions

#### Case A: A Day in the Life of a Young Married Pregnant Woman Living with HIV in Migori County Kenya

This narrative is based on data from qualitative and quantitative data collected from young (aged 18–24) pregnant and postpartum women, male partners, and other stakeholders as part of four studies conducted in southwestern Kenya over the period 2009–2019. The Maternity in Migori and AIDS Stigma (MAMAS) Study was a prospective mixed methods study of the effects of HIV-related stigma on pregnant women’s use of health services [49]. The Gender-Based Violence (GBV) Study aimed to address GBV in rural Kenya, with a special focus on pregnant women, and included piloting of GBV services, developing culturally appropriate training materials, risk stratification tools, referral protocols, and steps for local stakeholder engagement [50]. The Mother and Infant Visit Adherence and Treatment Engagement study (MOTIVATE Study) was a cluster randomized, 2 × 2 factorial, controlled trial to evaluate the individual and combined effect of mobile text messaging and community-based mentor mothers on maternal postpartum retention in care and ART adherence [51]. The Jamii Bora Study focused on the development and piloting of a home-based intervention for pregnant couples focusing on promoting couple relationship skills, couple HIV testing, prevention of mother-to-child transmission, and family health [52].

#### Case B: Street-Connected Adolescent Girl Seeking HIV Testing and Counselling in Kenya

This narrative draws on qualitative data collected from a study that sought to understand community perceptions of and responses to street-connected young people in Kenya that included 100 participants across 5 counties, of which 36 participants were street-connected young people aged 16 and 24 [53–55]. As well, this narrative draws on data and experiences from qualitative research that sought to understand street-connected children and youths’ sexual and reproductive health practices [56–59], and a series of pilot studies that sought to engage street-connected young people in the HIV prevention-care continuum [60–62].

#### Case C: Adolescent Girl Seeking STI Screening and Treatment Services

This case study is drawn from the Orphaned and Separated Children’s Assessments Related to their (OSCAR’s) Health and Well-Being study, a 10-year longitudinal cohort of orphaned and separated children and adolescents studying the effect of care environment on their health and well-being [63–65]. This case is also drawn from informal reports by numerous orphaned and separated adolescents over many years to the Kenyan investigators (personal communication: David Ayuku, Kenneth Ngure, Edith Apondi), and other efforts related to linking high risk adolescents including orphans to HIV prevention, testing, and treatment services [4, 7, 54, 59, 61, 63, 66–69].

#### Case D: HIV Testing Experiences Among a Young Refugee Woman in a Refugee Settlement

This narrative draws from qualitative data collected from young refugees aged 16–24 in Bidi Bidi settlement, Uganda in 2020. The Ngutulu Kagwero (Agents of Change) study was a mixed-methods study aimed at understanding and addressing sexual and gender-based violence experiences, access to post-rape clinical care, and sexual violence stigma, among refugee youth in Bidi Bidi [70]. The qualitative phase with young refugee sexual and gender-based violence survivors (n = 58, mean age 20.9 years, standard deviation 2.17) included six focus groups and 10 individual interviews (3 focus groups and 5 interviews with young women, 3 focus groups and 5 interviews with young men), in addition to 8 in-depth individual interviews with elders (n = 4 women, n = 4 men) and 10 health care providers (n = 5: women, n = 5: men) [14, 70–72].

#### Case E: Young Ghanaian Man Who Has Sex with Men (MSM) Seeking HIV Partner Services in an Adult HIV Clinic

This narrative is based on qualitative research conducted in Ghana between 2011 and 2013 using focus groups of social networks of MSM and individual interviews with healthcare workers from clinic facilities in Accra, Ashanti and Eastern regions. The Kumasi & Accra Project to Prevent AIDS (KAPPA) was a mixed-methods study that examined multi-level (social group-level and healthcare system-level) influences on HIV prevention among MSM in the three regions of Ghana with the highest prevalence of HIV [73–76].

### Analysis

We conducted a within and cross-case theoretical thematic analysis situated in the Health and Stigma Discrimination Framework [17, 77]. The framework can be applied to understand stigma processes across the social-ecological model and used to identify areas to improve the implementation of programmes, to understand how to address stigma, and what interventions, policies, and approaches may be appropriate to improve HIV prevention and treatment services in the context of stigma [17]. The framework consists of five domains (drivers, facilitators, stigma ‘marking’, manifestations, and outcomes) that break down the process of stigma and facilitate understanding the impacts of stigma on health and society. Our analysis focused on identifying major themes around the drivers and facilitators of stigma, understanding the application of stigma (i.e., ‘marking’), and manifestations of stigma. These correspond to the domains within the Health and Discrimination framework that are key areas for research, intervention, and programme monitoring. We then analyzed how these stigma domains operate and interact across levels of the social-ecological model (e.g., individual, interpersonal, community, organizational, and structural) to influence the uptake and delivery of HIV prevention and treatment services for adolescents as a contextual determinant [24]. As a result of this analysis, we determined key research questions and considerations of implementation science research to improve the HIV care continuum for adolescents in sub-Saharan Africa.

## Results

We present the following 5 narratives (cases A–E) that reflect the numerous and intersecting stigmas adolescents in Kenya, Uganda, and Ghana, experience that impact the uptake and delivery of HIV prevention and treatment services. Each case demonstrates how multiple social identities (e.g., gender and gender identity, sexual orientation, age, orphan status, refugee status, class-related, etc.) and health condition-related stigmas (e.g., HIV status, sexually transmitted infections, pregnancy, experience of sexual and gender-based violence) intersect and can contribute to worsening HIV-related outcomes for adolescents. The narratives establish how stigma operates across multiple levels of the social-ecological model (e.g., individual, interpersonal, community, organizational, and structural levels) and influences the uptake, delivery, and implementation of HIV services, interventions, and policies.

### Case A: A Day in the Life of a Young Married Pregnant Woman Living with HIV in Migori County Kenya

The following narrative includes experiences of intersectional stigma around HIV status and poverty, experienced by a young married pregnant woman living with HIV in rural southwestern Kenya. The woman described has multiple identities that are often stigmatized: she is young, poor, living with HIV, and fears intimate partner violence from her husband. These stigma experiences manifest at the individual, couple, family, community, and health facility levels.

Ruth (18 years old) wakes up early to prepare breakfast for her husband and do chores in the homestead, including preparing food for her husband’s mother and brothers who live in the same compound. Her husband is 10 years older than her and works in the transport industry and has another wife and home in a nearby town. She needs to ask him for money to buy food, but she worries because when she asks for money, he usually gets mad and complains he does not have enough to support everyone. Last month she went to the clinic and discovered on the same day that she was pregnant with her first child, and that she is HIV-positive. Although the nurses reassured her that she could take medicine to keep her healthy and protect the baby from getting HIV, she hasn’t had the courage to tell her husband or anyone else yet. She missed her ANC visit this month as the nurse insisted that she come back with her husband. From what she has heard around the village, husbands and their families can beat a pregnant woman and chase her from the home if she has HIV. “There are those who normally chase away their wives saying that they should just go, because he already thinks that the child is also having the disease. He will threaten to beat you up so your heart will be troubled because you have the disease, you are pregnant, and the man has chased you to go back to your home, all those will be painful. There is one story I heard about…that a man beat up his pregnant wife recently when she went to the clinic and was found with the virus.” (pregnant woman, age 22) [49]. Ruth knows that she cannot go back to her maternal home, as the family does not have enough resources to feed her and the child she is expecting [78]. She is concerned about how her in-laws would treat her if they learnt about her HIV diagnosis, as she had heard of cases where family members did not want to even share the same utensils as someone with HIV. She thought to herself, “You only disclose after knowing how you will survive” (pregnant woman, age 20) [79]. As Ruth goes about her load of household chores during the day, her heart is heavy. She doesn’t want to give birth in the hospital because she knows that women who deliver in a hospital are often assumed to be HIV-positive by other community members and may be treated badly by the doctors. She wants to talk to someone about what she is going through, and she feels her only options are to talk to her mother or her older sister who works in the capital city. She fears that her mother will yell at her for getting herself in this situation—a young pregnant woman with a husband who cannot support her and probably gave her HIV. Ruth decides to disclose her HIV-positive status to her sister, feeling that sharing her status would help her to be “free” and get some help for her troubles [80]. She has also heard from her neighbor that there are some people from the hospital who can help pregnant couples to test for HIV together and help with disclosing HIV status to each other [52]. With a heavy sigh, she looks for a private place to make the call. To Ruth’s surprise, her sister is actually supportive and understanding. Her sister says that she has friends the same age as Ruth who have HIV and they are able to live well and even have babies who were born without the disease. She advises Ruth to adhere to her HIV medications and disclose to others in the family as well, so that she can get help when she needs it and not be a burden to the family [80].

### Case B: Street-Connected Adolescent Girl Seeking HIV Testing and Counselling in Kenya

This case study portrays the intersectional stigma experienced by street-connected girls and young women when seeking HIV testing and counselling services in Kenya. Adolescent girls and young women connected to the streets have multiple intersecting stigmatized identities (e.g., gender, age, engagement in transactional and survival sex work, and street identities), which result in discrimination and several barriers to accessing HIV testing and counseling.

Faith is 15 years old and lives in a mabati (iron sheet) and mud room in a peri-urban slum with 4 other street girls. Before they moved here, they were sleeping on a shop’s veranda where they paid a watchman 50 Ksh ($0.50 USD) per day, but it didn’t always guarantee them safety at night. Over time the girls became friends and decided to combine their earnings to rent a room so they would be more secure at night. Faith and her friends come from different areas of Kenya, but they share a common story of migrating to the streets from a highly impoverished home that was rife with family dysfunction. During the day, they try to make ends meet and ‘hustle’, as most young people connected to the street do, through odd jobs and collecting recyclables, but as girls and young women they experience gender discrimination and are often told ‘they can’t work’. As a result, Faith and her friends find themselves begging and sometimes engaging in sex work in order to have enough money for food, clothing, toiletries, and rent [56, 61]. Labeled and stigmatized as ‘chokoraa’ (garbage pickers) by society for their street-involvement, as young women Faith and her friends face additional stigmatization for engaging in sex work [53]. The intersection of their street identity with sex work impacts their earnings as they are seen as ‘cheap’: “The men think they can get very cheap sex from us because we are vulnerable unlike the prostitutes. I think it is because we are desperate and need any money we can get.” Faith and her fellow street girls often help each other out and confide in each other about what happens to them, and recently Faith told her friend Sharon that she was offered an extra 100 bob to have sex without a condom. She needed the money and accepted even though she knew the risk. Sharon listened and understood Faith’s situation, provided her support, and told her she should get tested at the local youth clinic. Reluctant and scared knowing of her status and that she will lose money going to the clinic, she tells herself she will go the next morning. She tries her best to wash up the next morning with the limited water they have and put on some fresh clothing, but the people still treat her rudely when she boards the matatu, as they see her as a they see her as a thief and think she smells [53]. She already regrets going the way people are looking at her. After arriving at the health facility, the watchman at the gate looks her up and down and yells at her telling her she doesn’t belong here. Faith persists and says she is going to the youth clinic and the watchman finally relents and lets her pass and motions in the direction of the clinic. Faith has never been to this hospital and is unfamiliar with how to navigate the health system. The hospital is large, and she feels like everyone is looking at her and distancing themselves from her. She tries to ask some people for directions to the clinic but most just ignore her, but one kind young woman shows her the way to the clinic and helps facilitate getting her a test [60]. She is told to sit alone and wait, and Faith feels that just like on the matatu (public transportation), she is treated differently because of the way people see her and think about ‘chokoraa’ [53]. After waiting for over an hour, someone finally comes to get her, but they don’t understand each other well and the counsellor seems rushed and annoyed that her hands are dirty when doing the testing and has to use multiple swabs [54]. Faith is briefly lectured by the counsellor that she should get off the streets and go to school and go home to her parents like a ‘good girl’, and that girls her age shouldn’t be having sex and needing HIV tests. Faith can’t wait to leave this place and feels terrible. Her test was negative, but they tell her she needs to come back again in 3 months to re-test to make sure. Faith thinks to herself “no way am I coming back here, these people don’t understand my life”.

### Case C: Adolescent Girl Seeking STI Screening and Treatment Services in Nairobi

The following narrative speaks to the experiences of intersectional stigma and discrimination experienced by a young, female, economically marginalized, orphaned adolescent at high risk of sexually transmitted infections including HIV in Nairobi, and her challenges in accessing basic clinical services. Stigmas highlighted in this case include societal (patriarchal, gerontocratic culture), health facility (adolescent SRH needs), family (orphan status, adolescent SRH), individual (personal shame).

One day, Jemutai (17 years old, female) started experiencing stomach pain which overnight became so severe she decided she needed to visit a health facility first thing in the morning. The girl’s parents died of HIV leaving her no choice but to live with her aunt. As a young girl in a highly patriarchal, gerontocratic culture, she found she had little autonomy in decision making as she depended on her aunt financially who often mistreated her [64, 81]. Poverty-stricken with little education and no means of legal earning or employment and living in Nairobi, things don’t go so well. The aunt challenges her on her way out the door in the morning about where she’s going without having washed dishes or cleaned the house, and Jemutai feels she has little option but to lie and say she is going to see a friend. This immediately raises the aunt’s suspicions and she berates Jemutai for visiting men – which she actually did on occasion in exchange for money [63]. The aunt warns her that she will get a disease and wind up pregnant, becoming a further burden to the aunt who threatens to kick her out of the house. Eventually, Jemutai leaves the house, angry but relieved, and goes hurriedly to the local health facility, which it turned out had no adolescent services. She then went to a private hospital that specializes in STI’s (sexually transmitted infections) though mainly for adults. She is feeling anxious and depressed because she worries what people will think about her once her fears of an STI are confirmed. She manages to get to the facility and is seen by a nurse who asks her the purpose of the visit, but because Jemutai is a legal minor, inquires about where her parents or guardian are. Her question is followed by profound silence. She didn’t want her aunt to know she was coming to the facility let alone telling her what she suspects is ailing her. Then, because it is her first visit to this facility, she needs 200 KSH (approx. $2 USD) to get registered, which Jemutai does not have. As it is a private facility, she is turned away for lack of cash. Despondent, tired and in pain, Jemutai decides to go look for some small amount of money so that she can at least start treatment. Two days later, she comes back, symptoms worse than before. She manages to get registered and proceeds to see a clinician. As it becomes evident that the symptoms are consistent with a STI, the clinician questions her about why she waited so long to see a doctor, whether she is sexually active and in a marital relationship, whether she is in school, and where her parents are. When she explains her situation the doctor tells her she is not living as a good Christian and should feel shame. The clinician lectures her about the value of education and the morals associated with being a good Christian. The clinician eventually writes her a laboratory requisition and tells her to get tested. The clinician did not bring up HIV pre-exposure prophylaxis (PrEP), pregnancy, or family planning concerns. Without any other services, Jemutai goes to the lab which also requires money, which after some days she finds, gets tested, and receives a prescription for syndromic STI treatment. She is then referred for HIV testing which to her relief turns out negative. To add to the joy only she knew, she also tests negative for pregnancy. Disappointed and dissatisfied by how she was treated, she vows never to go back to the facility.

### Case D: HIV Testing Experiences Among a Young Refugee Woman in a Refugee Settlement

The case study presented reflects experiences of intersectional stigma regarding sexual violence, refugee status, gender, age, and HIV, experienced by a young refugee woman. These intersecting identities and related stigma shape exposure to sexual violence and experiences accessing post-exposure prophylaxis and HIV testing services.

Kiden (16 years old) woke up early to fetch water from the water pump. Many of the pumps are closed due to not enough water. The riverbeds are dry. The wait is so long, and it is so hot by the water pumps in operation during the day, that she decides that the best idea is to get up before sunrise. She washed her face, grabbed the two empty yellow jerry cans outside the door, and started on the long walk to the water pump that she had fetched water from yesterday. She did not expect that she would be attacked and raped on her walk. There were rumours going around that there were dangerous people hiding in bushes, but she did not know anyone that this had happened to and never imagined it would be her. She is shocked and afraid. What should she do next, she wondered. She knew that the community blamed girls who had been sexually assaulted and called them names, believing “the girls are always trying to seduce boys”. She also heard that anyone raped in the community was HIV-positive and may become pregnant. Kiden’s friend Tabu had been forced by her parents to marry the boy who raped her. Tabu was miserable, no longer allowed to go to school. Kiden was terrified about HIV, about possibly being pregnant, and most of all, about what would happen to her if anyone found out. She did not know the strange man who had attacked her, how could she be forced to marry such a person! She went home quietly and laid down, saying she was sick and unable to go to school or fetch water. Later in the day her friend Abdo came by to check on her: “Kiden, you were not in school, where have you been?” She looked down and told him she was hiding and started to cry, sharing him what had happened. Abdo offered to come with her to the clinic for HIV testing. There was a lot of people waiting on the wooden benches outside the clinic. She felt them staring at her. She didn’t know what to say when she went to the front, so Abdo came with her and said it was private. When she saw the healthcare worker she began crying. The healthcare worker explained to Kiden that she needed an HIV test, post-exposure prophylaxis pills, emergency contraception pills, and she needed to come back for another HIV test in 3 months. She was terrified. She did not know where she would hide all of these pills or what she would say to her parents. So, she said nothing. But the next day when she went to school, she felt all eyes on her and her people whispering she was spoilt and HIV-positive. She felt ashamed and suicidal. How did people know, and who had told them? Abdo promised it was not him, but it had likely been someone at the health clinic. She could not eat, she could not sleep, and days went by. Finally, her parents called her to ask her what was going on. When she told them, they asked why she had not come to them right away. Kiden described she was afraid they might make her marry the man, and that she still had to go for another HIV test in 3 months. Her parents shared that Tabu’s parents were worried she would not find another husband due to having experienced sexual violence, but they did not want to make permanent choices for her. They told her that they loved her, they want her to be healthy and happy, and her future would be bright. Also, she could take them with her when she went for the follow up HIV test for support.

### Case E: Young Ghanaian MSM Seeking HIV Partner Services in an Adult HIV Clinic

The narrative illustrates the operation of intersectional stigma at multiple levels (interpersonal and institutional). It also illustrates how intersectional stigma is deployed within healthcare facilities through social discourses of “normalcy” as well as how the experiences of intersectional stigma are highly individualized and can vary from person-to-person. The first patient is experiencing stigma at the intersection of his sexuality and his adolescent life stage; while the partner in the narrative is experiencing stigma at the intersection of his sexuality, adolescent life stage, perceived sexual motives, and non-conformity to perceived gender norms.

Abu, a 19-year-old man was diagnosed with HIV at birth. For the first 18 years of his life, he was in the specialty medical care of a pediatric infectious disease team. As he entered his teenage years, Abu’s pediatric team began to talk to him about sex and sexuality, with particular attention to helping Abu understand the benefits of sustained viral suppression. This included counseling regarding when he finds a bride and marriers her, he will be able impregnate her without onward transmission of HIV infection. When Abu turned 18, he was told by his pediatric care team that he was no longer eligible for services there and that he needed to establish himself as a patient with an adult HIV treatment team. Early in his adolescence, Abu understood that he had sexual and romantic attraction to other men. He is now at an age where his free time is more self-directed, and he has begun using this liberty to explore and satisfy his desires for same-gender sexual relations. In the process, he has met some other MSM who are now in his network of friends. He had heard from some of the older men in his network about an HIV clinic that is known to be MSM friendly. Abu transferred his care to the adult HIV clinic, but he missed his first two appointments because he arrived at the clinic late. On the third try he was finally successful at arriving on time for his appointment and was seen by the clinician, but he felt like the visit was about “HIV” not about ‘Abu.” When the clinician asked him about his intentions to marry and have children, he let team know that he was finding that he enjoyed the intimate company of men more than he did with women and that he was intending to find a boyfriend and maintain a relationship with him. Several members of the clinical team admonished him that he should “keep quiet” and not discuss those things because he is “too young to know what he wants.” As Abu was leaving the clinic, he noticed that some people were going into exam rooms in pairs. He asked about it and the nurse let him know that they provide partner counseling and services, and the clinic is supportive of partners attending visits together, even if the partner does not have HIV. At the next visit, Abu comes in with his new boyfriend, Salifu (but who prefers to be called “Sali”). Abu and Sali have been having sex together and Sali, wants to learn about HIV pre-exposure prophylaxis (PrEP). The receptionist alerts the clinical team that Abu has arrived for his visit but expresses concern that Abu arrived with man is who is looking and behaving very feminine and womanly. She suggests that Abu and Sali be moved out of the general waiting room into an empty exam room to wait there because some of the patients are in the waiting room with their families, including children. The clinical team agrees. When the clinician begins the visit, Abu is frustrated and asks why they have been segregated. The clinician responded that Abu had disrespected the clinic by bringing in such a flamboyant partner. Abu defended his partner and said there is nothing wrong with Sali’s manner or appearance, to which the clinician replied that “If you can’t see the problems with a man acting like a woman—in a public place, it shows your immaturity and ignorance about life”. When Sali asks about PrEP he is told that PrEP requires daily adherence and by looks of the ways he is dressed, and acts demonstrates that he does not have self-discipline to use PrEP. He turns to Abu and states: “I don’t blame you, Abu. You were born with HIV. It is not your fault; but this person wants the PrEP so that they can be wild and just have more sex with you and probably others”. Abu and Sali are disappointed in the clinician’s position, but they persist in trying to get their health needs met. Abu mentions a recent conversation in an online chat group about sero-positioning and wanted a professional clinical opinion of whether they can reduce Sali’s risk of acquiring HIV during sex if he performs as the bottom (receptive partner) and Sali performs as the top (insertive partner). The clinician becomes disgusted and says “Wow, so your plan is for the woman to now penetrate the man? Foolish child.” Sali interjected: “we are BOTH men” and Abu added: “We are not children.”

Our within and cross-case analysis identified 5 major themes and 18 sub-themes, corresponding to domains within the Health and Discrimination Framework [17].

Table 2 summarizes the major themes of stigma drivers, stigma facilitators, and stigma ‘marking’ and associated sub-themes. Our analysis assesses how stigma drivers, facilitators, and stigma ‘marking’ operate across the social-ecological model [24], describes how these stigma domains impact the uptake and delivery of HIV services, and identifies research questions and implications for implementation science research.

**Table 2:** A social-ecological approach to understanding how stigma drivers, facilitators, and stigma ‘marking’ impact the uptake and delivery of HIV services and implications for implementation science research to address stigma

Health stigma and discrimination framework domains	Cases theme within	Operating across social–ecological levels	Examples of the impact of stigma on the uptake and delivery of HIV services from selected cases	Considerations for addressing stigma as a contextual determinant of implementation and uptake of HIV prevention and treatment services
Stigma drivers				
Fear of infection	B, C, D	Individual, Interpersonal	Faith is reluctant to seek care and scared to know her status. This demonstrates how stigma may reduce the likelihood that adolescents will engage in HIV testing and counseling	When designing approaches to HIV testing and counseling and HIV prevention education, there is a need to consider the strength of fears and other emotional factors associated with stigma, which affect the individual and interpersonal relationships, and impact the uptake of HIV services
Fear of social ramifications	A, C, D,	Interpersonal, Community	Kiden fears the social ramifications of experiencing SGBV and HIV-positive status and being forced to marry the boy who raped her. For Kiden, this led her to hide what had occurred from her parents and demonstrates how stigma drivers may impact the uptake of follow-up care and reduce disclosure	These cases demonstrate the crucial importance of addressing contextual social-cultural drivers *outside* of the health facility, including the family, that impact the uptake of and access to services
Fear of economic ramifications	A, B, C	Interpersonal, structural	Ruth fears the economic ramifications of HIV disclosure and being outcast by her in-laws and chased from home, without the economic resources for her maternal family to care for her and her child, she is unsure how she would survive. For Ruth, this fear impacts her uptake of ANC services	These cases demonstrate the need to address how factors such as the fear of economic ramifications operate across levels of the social-ecological model when implementing interventions to improve the uptake and delivery of HIV services to adolescents
Social judgment	A, C, D, E	Interpersonal, Community, Organizational	When Abu arrives at the clinic with Sali, the receptionist expresses social judgment about Sali’s gender identity, driving stigmatizing practices when they are told to wait in a separate area. Social judgment within the health facility impacts the delivery of HIV prevention services for Abu and his partner	These cases demonstrate the importance of ensuring health care providers and facility staff are trained and sensitized to work with adolescent key populations, as social judgment within the facility is a barrier to effectively implementing HIV services
Blame	A, C, D	Individual, Interpersonal, community	Kiden knows that the ‘*community blamed girls’* who have experienced SGBV. Blame can reduce the likelihood that adolescents will engage in HIV testing and counseling or disclose their status, as friends, family, and community members will assign them responsibility and fault for acquiring HIV	These cases demonstrate the importance of considering how assigning fault and responsibility to an individual is internalized and may act as a barrier to care and the uptake of HIV services. Interventions need to consider the interpersonal and community-level aspects of blame as a contextual barrier to the uptake and delivery of HIV services
Prejudice	A, B, C, D, E	Individual, interpersonal, community, organizational	Faith engages in sex work due to prejudice expressed in the patriarchal street subculture that girls/women ‘*can’t work’*. Because of her engagement in sex work on the street she is not considered to be a ‘*good girl’* by the counselor. The preconceived notion by healthcare providers can impact patient-provider interactions and the delivery of HIV services to adolescents	These cases demonstrate how prejudice operates across levels of the social-ecological framework and can impact the uptake and delivery of HIV services in several ways. At the health facility level, training facility staff and ensuring the facility is inclusive and provides equitable services to all regardless of their social identities is a vital consideration when implementing programmes, services, and policies
Stereotypes	A, B, C, D, E	Individual, interpersonal, community	The clinician stereotypes Sali believing that he will be unable to adhere to PrEP due to his appearance, thus impacting Sali’s ability to receive PrEP and the delivery of effective HIV services from the clinician	Widely held beliefs about groups of individuals are often difficult to shift and can have a major impact on the uptake and delivery of HIV services across multiple levels of the social-ecological model, thereby likely requiring multi-component interventions to improve implementation outcomes
Stigma facilitators				
Social support	A, B, D, E	Interpersonal, Community	Ruth receives social support from a neighbour about HIV testing and disclosure for couples. As well, she receives an understanding and helpful response from her sister that encourages her that her family will be there for her, and she is not a burden. Social support from friends, peers, family, and within the community can mitigate the stigma process, and improve the uptake of HIV services, such as medication adherence and retention in care for Ruth	These cases have shown how the presence of strong positive social support from family, friends, peers, and social networks is an important component to improve the uptake of HIV services. Social support, peers, and social networks may play an important role in bridging contextual barriers that influence the implementation and uptake of HIV services
Gender norms and equality	A, B, C, D, E	Individual, interpersonal, community, structural	The clinician seeing Abu and Sali becomes disgusted with them around their sexual identities and decision-making around receptive versus insertive sex practices, referring to their masculinity and femininity. The clinician treats them with disrespect resulting in a stigma facilitator impacting the delivery of HIV care and prevention to Abu and his partner	The impact of gender norms and equality is seen across cases and occurs across multiple levels of the social-ecological model. The expectations of women, men, and gender-diverse individuals act in society are an important contextual aspect that can affect the implementation of interventions, approaches, and policies to improve the uptake and delivery of HIV prevention and treatment. Stigma toward same-sex sexual practices needs to be addressed in healthcare provider training
Cultural norms	A, B, C, D, E	Individual, interpersonal, community, structural	Jemutai is lectured by the clinician about being a ‘good Christian’. Stigma enacted by clinicians reinforces social-cultural stigma around adolescent sexuality and health, instead of creating healthy and positive norms around adolescent SRH and sexual education. Reinforcing shame around sexual activity can lead to high-risk practices to hide engagement in sex and reduce likelihood of re-engaging in care for HIV testing and counseling	Cultural norms are another contextual barrier that operate across multiple levels of the social-ecological model. Deeply held cultural beliefs by health facility staff and healthcare providers can impact the delivery of evidence-based HIV services
Legal environment	C	Structural	Jemutai has difficulty accessing services on her own given her limited financial resources and status as a minor coming for testing without an adult guardian	This case demonstrates the importance of identifying legal and policy level issues that affect the implementation of HIV services
Stigma ‘marking’				
Homelessness	B	Community, organizational, individual	As a result of stigma marking, stigma is applied to Faith targeting her street identity, and has an impact on health systems responsiveness, and her interactions with individuals in the health facility, and therefore the uptake of delivery of HIV services	This case shows the importance of addressing deeply held beliefs and attitudes healthcare providers and facility staff have about groups of individuals who have been ‘marked’ or labeled by society when implementing evidence-based interventions to improve uptake and delivery of HIV services
Sexual stigma	C, D, E	Community, individual	As a result of stigma marking, stigma is applied to both Abu and Sali due to HIV status and sexual orientation, impacting the patient-provider interaction, and the delivery of HIV services	This case demonstrates the importance of addressing deeply held beliefs and attitudes healthcare providers and facility staff have about groups of individuals who have been ‘marked’ or labeled by society when implementing evidence-based interventions to improve uptake and delivery of HIV services
HIV stigma	A, D	Community, interpersonal	As a result of stigma marking, stigma is applied to Kiden by her peers at school and she is marked as ‘spoilt’ and HIV-positive. This may facilitate internalized stigma, shame, and act as a barrier to the uptake of HIV services for Kiden	Stigma marking impacts the uptake and delivery of HIV services outside and within the health facility, and contextual barriers to implementation operate at multiple levels of the social-ecological model that are vital to consider

*SGBV* sexual and gender-based violence; *ANC* antenatal care; *PrEP* pre-exposure prophylaxis

### Stigma Drivers

Stigma drivers are negative and pre-existing factors that enable the stigma process prior to the application of stigma to an individual or community. We identified the sub-themes of fear of HIV infection, fear of social and economic ramifications, social judgement, blame, prejudice, and stereotypes as the drivers of intersectional stigma in our narratives. For example, the fear of social ramifications was present in the majority of narratives, as explained using the case A of Ruth: *From what she has heard around the village, husbands and their families can beat a pregnant woman and chase her from the home if she has HIV*. The fear of social ramifications in this example demonstrates how stigma drivers operate across multiple levels of the social-ecological model. In this case there is an interplay between individual (e.g., Ruth’s husbands’ knowledge and attitudes), interpersonal (family relationships) and community-levels (cultural norms, values, and attitudes in the village). Stigma drivers impact Ruth’s uptake of ANC and skilled delivery services, which are crucial for the Prevention of Mother to Child Transmission (PMTCT). Our example demonstrates the need for implementation science research to consider social-cultural and interpersonal contextual factors, i.e., the outer context, operating outside of the health facility that act as barriers to the uptake of HIV services.

### Stigma Facilitators

Positive and negative factors, including social and community norms, may mitigate or exacerbate the stigma process. We identified sub-themes around social support, gender norms and equality, cultural norms, and legal environment that influenced the stigma process across cases. Inequitable socio-cultural gender norms and traditions were negative influences facilitating the stigma process in all cases. Social support was a strong positive influence on mitigating the stigma process and improving the uptake of HIV prevention and treatment services by adolescents across four cases. For example, in case B, Faith confides in her friend Sharon who listens without judgment and provides support and guidance to seek care. Social support can be an important positive influence that operates at the interpersonal (e.g., Sharon’s friendship) and community levels (e.g., within the street social network of girls) of the social-ecological model. In this case, social support encouraged the uptake of HIV testing and counseling. Social support, particularly from peers, may play a vital role in bridging contextual barriers that influence the implementation and uptake of evidence-based HIV prevention and treatment interventions.

### Stigma ‘Marking’

Stigma marking is the process in which stigma is applied to individuals or groups. Our analysis identified that each case had multiple and intersecting stigmas, which impacted the uptake and delivery of HIV prevention and treatment services. Stigma was associated with health related-conditions including HIV-status, sexual and gender-based violence, sexual health, and pregnancy as well as multiple and intersecting social identities of the adolescents in the cases including gender, age, refugee status, poverty, homelessness, sexual orientation, and class-related stigmas. The application of stigma marking is the precursor to the manifestation of stigma in our cases.

Table 3 summarizes the major themes of stigma practices and stigma experiences. Our analysis exhibits how stigmatizing behaviours (e.g., exclusion, avoidance, gossip) and discriminatory attitudes result in stigmatizing experiences (e.g., experienced stigma and discrimination and anticipated and internalised stigma) for adolescents and together impact the uptake and delivery of HIV prevention and treatment services.

**Table 3:** A social-ecological approach to understanding how stigma practices and stigma experiences impact the uptake and delivery of HIV services, operate across levels of the social–ecological model, and implications for implementation science research to address stigma

Health stigma and discrimination framework domains	Cases theme within	Operating across social–ecological levels	Examples of the impact of stigma on the uptake and delivery of HIV services from selected cases	Considerations for addressing stigma as a contextual determinant of implementation and uptake of HIV prevention and treatment services
Stigma practices				
Stigmatizing behaviours	A, B, C, D, E	Individual, interpersonal, community, organizational	Faith is told to sit alone to wait (exclusion) and is treated differently from others within the clinic when attempting to access HIV testing. Faith recognizes she is experiencing discrimination in the clinic due to her identity as a street youth. As a result, Faith will not return for follow-up HIV testing and counseling, thus influencing the access to and acceptability of services	Stigmatizing behaviours can occur outside or within the health facility, and across multiple levels of the social-ecological model to impact the uptake and implementation of HIV prevention and treatment services. Multi-component, targeted, and tailored interventions need to consider how to address stigmatizing behaviours affecting HIV services
Discriminatory attitudes	A, B, C, D, E	Individual, interpersonal, community, organizational	The clinical team chide Abu telling him he is too young to know what he wants and that he should ‘keep quiet’ about his sexual identity. The intersecting stigmas of Abu’s age and sexual orientation underpin the manifestation of discriminatory attitudes by clinical staff. By invalidating Abu’s identities and healthcare needs, the health system lacks responsiveness and HIV services are not delivered appropriately to meet his needs	Discriminatory attitudes within the organization as well as those held by individual providers and facility staff can act as barriers to the implementation and delivery of evidence-based HIV services, intervention, and policies and need to be addressed to improve the adoption and implementation of programmes and policies
Stigma experiences				
Experienced stigma and discrimination	A, B, C, D, E	Individual, interpersonal, community, organizational, structural	Sali experiences discrimination when they are refused PrEP on the basis of their gender and sexual orientation leaving their health needs unmet and increasing HIV risk behaviours for Sali	Experiences of stigma and discrimination operate at all levels of the social-ecological model to impact the uptake and delivery of HIV services. There are opportunities for implementation science to understand and address how stigma is affecting HIV services
Anticipated stigma	A, B, C, D	Individual	Ruth anticipates stigma from the community if she gives birth at the local hospital as women who give birth in-hospital are assumed to be HIV-positive by the community. The anticipation of stigma in this case impacts the uptake PMTCT and having an in-hospital birth which would reduce the likelihood of mother-to-child transmission and improve childbirth outcomes for mother and child	The anticipation of stigma by adolescents may hinder their uptake of HIV services. Implementation science research should consider how to reduce and mitigate the effects of anticipated stigma on the uptake of HIV services
Internalised stigma	D	Individual	Kiden feels ashamed and suicidal when it became apparent her peers knew about her rape and made assumptions about her HIV status. Internalised stigma can reduce the likelihood of remaining engaged and retained in HIV care	Internalised stigma can act as a barrier to the uptake of HIV prevention and treatment services for individuals. Implementation science research should consider how to reduce and mitigate the effects of internalised stigma on HIV care

*PMTCT* Prevention of mother-to-child transmission

### Manifestations: Stigma Practices

We identified sub-themes around stereotypes, prejudice, stigmatizing behaviours, and discriminatory attitudes; however here we discuss only the impact of stigmatizing behaviours and discriminatory attitudes as we have examined stereotypes and prejudice as drivers of the stigmatization process. Stigmatizing behaviours and discriminatory attitudes were pervasive across cases and manifested in similar ways across each case regardless of different intersecting stigmas experienced by adolescents in our narratives. Stigma practices occurred outside and within the health facility and across social-ecological levels. For example, Abu and Sali are moved outside of the waiting room to an empty exam room at the clinic. Here stigmatizing behaviours may be operating at the individual (e.g., attitudes and behaviour of the receptionist) and organizational levels (e.g., workplace policy, organizational values, lack of healthcare provider stigma reduction training) to impact the delivery of HIV services to Abu and his partner. Once stigma has manifested, it has important implications for implementation science research to consider the effects of stigmatizing behaviours by facility staff and management as bottlenecks to the adoption, uptake, and implementation of evidence-based HIV services and policies by communities and practitioners.

### Manifestations: Stigma Experiences

We identified sub-themes around experienced stigma and discrimination, anticipated, and internalised stigma. The cases show a range of experiences of stigma and discrimination among adolescents based on their intersecting social identities. For example, Faith experiences stigmatization and discrimination when trying to access and navigate receiving HIV testing and counseling. In the case of Faith, she experiences stigma and discrimination in the community (e.g., on public transportation), at organizational levels (e.g., from the facility security guard), and at the individual/interpersonal level (e.g., in her interaction with the healthcare provider). Experiences of stigma and discrimination can impact the uptake and delivery of HIV prevention and treatment services through different pathways and levels of the social-ecological model, indicating how implementation science research is needed to understand and address how stigma is affecting HIV services.

We generated a series of case-related research questions and through our analysis identified intersectional stigma-informed implementation science research opportunities (Table 4). The research questions emphasize avenues for considering the impact of intersectional stigma across the HIV prevention-care cascade for diverse groups of adolescents and demonstrate how intersectional stigma may be addressed at different levels of the social-ecological model. For each case-related research question we identified corresponding opportunities for intersectional stigma-informed implementation science research. These opportunities explore how implementation science research can facilitate an understanding of how stigma-related barriers may be addressed along the HIV prevention-care continuum and identify the need to examine and assess the acceptability, appropriateness, feasibility, cost, and sustainability of interventions, practices, and policies to do so.

**Table 4:** Case-related research questions and intersectional stigma informed implementation science research opportunities

Case-related research questions	Intersectional stigma-informed implementation science research opportunities
Interpersonal level	
How can health services engage male partners of young pregnant women living with HIV in a positive, supportive, and non-coercive way that does not put women at risk of further stigmatization and violence?	Examine the acceptability and appropriateness of health services engaging male partners in HIV and ANC care in the context of stigma
How can positive supportive relationships (siblings, friends, other peers, family, neighbours etc.) be leveraged to help young people to support each other, resist stigma, and deliver HIV education and services?	Assess the appropriateness and feasibility of leveraging the positive supportive relationships among peers to reduce stigma and deliver HIV education and/or services
How can disclosure support be implemented in a way that protects the privacy, preferences, and rights of young people living with HIV, while also protecting the health of others in their family?	Evaluate the appropriateness of disclosure support with providers, patients, and families
How can disclosure support for HIV-positive results protect privacy, rights of young people with HIV, with particular attention to the ways that HIV-positive serostatus can lead to additional experiences of sexual and gender-based violence for young refugee women?	Determine the feasibility of disclosure practices with refugee women that seek to protect privacy and rights and understand unintended consequences of disclosure in specific contexts
Organizational level	
What aspects of health facility environments contribute to young people’s experiences of stigma and discrimination in healthcare settings? How could they be redesigned?	Determine health facility contextual barriers and facilitators of stigma and identify evidence-based practices and policies to reduce stigma and discrimination and improve HIV care
How can interventions to reduce stigma be adopted and implemented across the health facility to address stigma that starts at the door?	Determine health facility contextual barriers and facilitators of stigma and identify evidence-based practices and policies to reduce stigma and discrimination and improve HIV care
How can health service providers be trained to provide youth friendly and non-stigmatizing HIV cascade services for both adolescent girl and adolescent boys in complex contexts, such as humanitarian crises, or within settings with deeply held religious and moral values around adolescent sexuality and reproductive health?	Identify evidence-based training interventions focused on gender, HIV, and adolescents and determine feasibility of use within different contexts
How do you implement SRH/HIV interventions (testing, treatment, prevention) and reduce intersectional stigma in contexts where adolescent sexuality and reproductive health is stigmatized?	Examine the acceptability, appropriateness, and feasibility of implementing evidence-based interventions, practices, and policies to reduce intersectional stigma and increase HIV testing, prevention, and treatment with adolescents
How can HIV services be tailored and trauma-informed for adolescents in humanitarian contexts to ensure confidentiality, and encourage attendance either alone by adolescents or accompanied by the adolescent’s preferred support persons (e.g., friend, family)?	Assess the appropriateness and feasibility of trauma-informed adolescent-friendly HIV services within humanitarian contexts
Community level	
How do evidence-based interventions for stigma reduction need to be adapted to address stigma experienced by street-connected young people at the community level that act as barriers to seeking care and healthcare services?	Identify evidence-based intersectional stigma reduction interventions that can be adopted or adapted for use with street communities to improve the uptake of HIV services
What are the best means and messages for community sexual and reproductive health information for adolescents that reduce stigmatization of adolescent engagement in SRH?	Determine the acceptability, appropriateness, and feasibility of evidence-based community SRH health promotion interventions with adolescents in specific contexts
How can strengths-based approaches be applied with adolescent key populations to reduce stigma and facilitate increasing the uptake of HIV testing and counseling and use of PrEP?	Determine the appropriateness and feasibility of using strengths-based approaches with adolescent key populations to reduce stigma and improve the uptake of HIV testing and counseling
Structural/policy level	
How can structural interventions for adolescents (self-efficacy, economic empowerment) be incorporated/integrated into SRH and other health service delivery programs to reduce stigma?	Determine the feasibility and cost of incorporating structural interventions into health services delivery to improve adolescent engagement in SRH and reduce stigma
Multi-level questions	
How can multi-level intersectional stigma reduction (addressing adolescent SRH stigma, refugee stigma, inequitable gender norms, HIV-related stigma, sexual violence stigma) be developed and implemented with meaningful engagement of adolescent refugees?	Evaluate the acceptability, appropriateness, and feasibility of multi-level intersectional stigma interventions designed with adolescent refugees
What are the barriers and facilitators influencing the implementation of HIV partner services in healthcare facilities? What are the healthcare system-level influencing factors? What are the influencing factors at the interpersonal level of the patient and provider?	Identify contextual factors that influence the implementation of HIV partner services across levels of the social-ecological model
What is the range of stigma-reduction interventions that can be used address the barriers to implementation of partner services in young MSM? What stigma-reduction interventions can be combined to address the intersectional stigma on the implementation of PrEP and partners services for young MSM?	Identify what evidence-based stigma reduction intervention can be adopted to improve partner services for young MSM and determine what evidence-based interventions need to be adapted to address stigma around PrEP and partner services for young MSM

Abbreviations: sexual and reproductive health (SRH); men who have sex with men (MSM)

## Discussion

Our multiple-case study analysis identified several commonalities across cases, despite contextual differences, in how intersectional stigma impacts the uptake and delivery of HIV prevention and treatment services for adolescents in three exemplars from sub-Saharan African countries. Common across all cases, stigma drivers, particularly fear of social and economic ramifications and social judgement and blame by peers, family, community members, and health facility staff operated to negatively impact the uptake and delivery of HIV services for adolescents. Social, cultural, and gender norms that stigmatize adolescent sexuality and same-sex sexual relations and promote gender normative standards were key facilitators of stigma across cases and contexts. Notably, strong social support from peers and family members had a positive influence on mitigating the effects of stigma in all cases. These findings demonstrate the need to consider and address stigma drivers and facilitators across social–ecological levels when planning and implementing services, interventions, and policies to improve the uptake and delivery of HIV services for vulnerable adolescent populations.

Our findings demonstrated a range of intersectional stigmas experienced by adolescents in examples from three sub-Saharan African settings, and how multiple and intersecting stigmas affect HIV-related outcomes for adolescents. Manifestations of stigma (stigma practices and experiences) directly influenced the uptake of HIV testing and counseling, PMTCT, the use of PrEP and PEP, HIV disclosure, and increased the risk of experiencing violence, and reduced the likelihood of adolescents being retained in care across cases. Discriminatory attitudes, stigmatizing practices, and anticipated stigma acted as barriers to accessing care and as deterrents for remaining engaged and retained in care across cases. Stigma practices and discriminatory attitudes affected the delivery of HIV services by healthcare providers and influenced health systems responsiveness to adolescents seeking care [82]. Together the cases demonstrated that adolescents’ right to health and the quality, availability, and acceptability of healthcare is influenced by stigma as well as laws and policies concerning requiring consent from minors when accessing care. Stigma has serious consequences for the health and well-being of adolescents at-risk of acquiring or living with HIV in these contexts. These findings demonstrate the vital need for multi-level and multi-component evidence-based interventions to meaningfully reduce stigmatization and discrimination that impact the uptake and delivery of HIV services among adolescent populations.

Our multiple-case analysis identified several cross-cutting implications for implementation science research and generated research questions regarding interventions that seek to reduce stigma drivers as well as to mitigate harmful attitudes and norms. Implementation science offers the ability to identify, understand, and address intersectional stigma as a contextual determinant to the uptake and implementation of evidence-based practices and policies aimed at reducing stigma and improving the uptake and delivery of HIV services for adolescents [38, 42, 47]. To date, few studies have investigated the adoption, appropriateness, cost, fidelity, penetration, or sustainability of interventions seeking to reduce stigma in LMICs [42]. We have generated several case-based research questions and developed intersectional stigma informed implementation science research opportunities to fill this gap across multiple levels of the social-ecological model to address intersectional stigmas impacting the uptake and delivery of HIV services. (See Table 4).

This work makes an important and valuable contribution to the literature identifying the impact of intersectional stigma on the implementation of HIV prevention, testing, and treatment services for adolescents in sub-Saharan Africa. Stigma represents a significant barrier to the utilization and delivery of HIV services among adolescents [14–16]. As adolescents continue to be disproportionately affected by HIV and less likely to engage in HIV prevention practices [4, 5, 7, 8], addressing intersectional stigma as a contextual barrier to the uptake of delivery of HIV services, interventions, and policies is critical. Several evidence-based interventions exist to prevent and treat HIV among adolescents [36], yet HIV-related stigma is likely a key factor hindering the implementation of adolescent HIV prevention and treatment for adolescents in sub-Saharan Africa [83]. Intersectional stigma informed implementation science research represents an avenue to address stigma as a contextual barrier and enhance the uptake of evidence-based interventions.

This work has limitations and strengths. This multiple-case study drew on data collected from three sub-Saharan African countries and therefore may not be representative of experiences of adolescents across the continent in different countries and contexts. Indeed, rooted in qualitative traditions, case studies do not aim for representativeness or generalizability. Further, we generated five cases that reflected the intersectional stigmas experienced by AGYW and key adolescent populations; however, these cases are not exhaustive and given the diversity of intersectional stigma experiences our findings may not be representative of the multiple intersecting stigmas experienced by adolescents in sub-Saharan Africa. Despite these limitations, these exemplars present a diversity of cases spanning several countries and adolescent populations generated from multiple source studies and experiences and contribute to understanding the myriad forms of intersectional stigma that shape HIV outcomes. Our approach of applying the Health Stigma & Discrimination Framework across these diverse cases can be used by other researchers and practitioners to better understand and address stigma in diverse contexts and populations. The discussion of implementation science considerations generated from this approach to analysis, and specifically our exemplars of intersectional informed implementation science research opportunities, can inform future implementation science research in the context of intersectional stigma and HIV.

## Conclusion

Intersectional stigma is a key implementation determinant influencing the uptake and delivery of HIV prevention, testing and treatment services for adolescents across the HIV prevention and care continuum in sub-Saharan Africa. Implementation science research affords opportunities to understand and address how stigma acts as a contextual barrier to the adoption, uptake, implementation, penetration, and sustainability of HIV prevention and treatment interventions for adolescents.

## Data Availability

On reasonable request.
